# Supplementary material for: Shared Decision-Making at the Intersection of Disability, Culture, and Language Accessibility: An Educational Session for Medical Students
Source: MedEdPORTAL. 2024 Apr 30;20:11396. doi: 10.15766/mep_2374-8265.11396 (PMC11058081; doi:10.15766/mep_2374-8265.11396)
Supplement: Supplementary file 1 — Facilitator Guide.docxQuestions for Panelists.docxHearing and Listening.mp4Disability, Culture & Language Accessibility.pptxShared Decision-Making Lecture.mp4Session Guide.docxStudent Guide.docxSession Evaluation Tool.doc [file mep_2374-8265.11396-s001.zip › B. Questions for Panelists.docx]

Questions for Panelists for Disability and Communication Panel

*Faculty members, utilize this as a comprehensive guide for orienting the Disability and Communication Panelists. The document has been directly shared with the panelists, accompanied by a dedicated 30-minute orientation session preceding the panels. We strongly suggest establishing community partnerships with various disability organizations and reaching out to individuals with disabilities interested in sharing their healthcare experiences.*

Learning Objectives for people with disabilities (PWD) and their family members Panel:

- Describe the ability of all persons, regardless of disability to provide valid consent that reflects respect and self- determination.

Emphasis on:

- Informed, valid consent and the assumptions that physicians may make when taking care of a person with a disability
- Asking questions, shared decision-making

General points for panelists

- Students understand that these sessions may include your private information and that they should not share that information in a way that could identify you—just like when you go to the doctor
- You do not have to answer any questions you feel uncomfortable with, and don’t even have to give your real name
- As you prepare to participate, think about one or two key points you would want a student to remember for the rest of their career

Questions:

1. Please introduce yourself (30-60 seconds)
   1. What is your relation to the community of people with disabilities
2. Share any positive or negative experiences you’ve had with your loved one when seeing a physician, specifically regarding their disability?
3. How has the physician involved you in the decision-making process?
4. What styles of communication do you value in healthcare settings? Or does your young adult respond the best to?
5. What would you want medical providers to know when advising patients and families of patients who have disabilities?
6. Is there anything/ any investigations/ questions you think are consistently left out when you are in a healthcare setting?
