# Supplementary material for: Shared Decision-Making at the Intersection of Disability, Culture, and Language Accessibility: An Educational Session for Medical Students
Source: MedEdPORTAL. 2024 Apr 30;20:11396. doi: 10.15766/mep_2374-8265.11396 (PMC11058081; doi:10.15766/mep_2374-8265.11396)
Supplement: Supplementary file 1 — Facilitator Guide.docxQuestions for Panelists.docxHearing and Listening.mp4Disability, Culture & Language Accessibility.pptxShared Decision-Making Lecture.mp4Session Guide.docxStudent Guide.docxSession Evaluation Tool.doc [file mep_2374-8265.11396-s001.zip › F. Session Guide.docx]

**Shared Decision Making at the Intersection of Disability, Culture, and Language Accessibility**

**Learning Objectives:**

At the end of the Learning Community, students should be able to:

1. Identify that non-English language preference can act as a social determinant of health (for example in individuals who communicate using American Sign Language).

2. Apply elements of the medical and social models, practicing cultural humility, as appropriate in the context of Deaf culture.

3. Apply the key components of valid consent using a shared decision making framework.

4. Describe the ability of all persons, regardless of disability to provide valid consent that reflects respect for self- determination.

*Keywords:* *Shared decision making, Disability/Deaf Culture, Cochlear Implant, Self-determination*

**Pre-Session Self-Directed Learning Activities:**

(30 min)

**Required Readings/Videos:**

1. Hearing and Listening: Health Equity, Deaf Culture, and Communication for Healthcare Professionals (6 min)
2. Shared Decision Making Lecture (11 min)
3. Disability, Culture, and Language Accessibility: Terms and Communication Skills PowerPoint (10 min)

**Recommended Readings/Videos:**

1. McCullough LB, Chervenak FA. Informed consent. Clin Perinatol. Jun 2007;34(2):275-85, vi. doi:10.1016/j.clp.2007.03.005
2. Benedict BS. Deaf Culture & Community. 2020;Communication Considerations A to Z™. https://www.handsandvoices.org/comcon/articles/pdfs/deafculture.pdf

**Learning Community Session Content:**

- Patient Scenario Discussion and Role-Play Exercise in Small Groups (45 min)
- (Optional) Disability Panel in Large Groups (45 min)

**Post-Session Self-Directed Activities:**

- Post-Session Evaluation (5 min)
