# Supplementary material for: Shared Decision-Making at the Intersection of Disability, Culture, and Language Accessibility: An Educational Session for Medical Students
Source: MedEdPORTAL. 2024 Apr 30;20:11396. doi: 10.15766/mep_2374-8265.11396 (PMC11058081; doi:10.15766/mep_2374-8265.11396)
Supplement: Supplementary file 1 — Facilitator Guide.docxQuestions for Panelists.docxHearing and Listening.mp4Disability, Culture & Language Accessibility.pptxShared Decision-Making Lecture.mp4Session Guide.docxStudent Guide.docxSession Evaluation Tool.doc [file mep_2374-8265.11396-s001.zip › G. Student Guide.docx]

**Shared Decision Making at the Intersection of Disability, Culture, and Language Accessibility: Patient Scenario Discussion**

Students, kindly access this document within your designated small groups to engage in group discussion and role-play.

Learning objectives

1. Apply elements of the medical and social models, practicing cultural humility, as appropriate in the context of Deaf culture.
2. Apply the key components of valid consent using a shared decision-making framework.

Case

Martha and Joe Smith are presenting at your clinic today with their newborn Ellie, who has been diagnosed with profound bilateral deafness. You are their primary care physician, who has already taken care of the Smith’s older children. After birth, Ellie did not pass the newborn hearing screening which prompted the standard need for confirmation, a more intensive evaluation with the audiologist. Over 90% of children with congenital hearing impairment are born to hearing parents.^1^ However, both Martha and Joe are Deaf individuals, who use American Sign Language (ASL) and are well integrated in the Deaf cultural community. You have purposefully allocated extra time for this appointment with the Smiths in order to discuss all of their options, and create the team of specialists who will follow Ellie. Of course, you provide an ASL interpreter as requested by the parents and use the teach-back method to confirm their understanding of your conversations. At her age and with her hearing loss, Ellie is a good candidate for the cochlear implant. In preparation for their visit, you took a few minutes to review the cochlear implant procedure.

1. In groups of two, utilizing the information listed below, practice acting as a parent and a physician in obtaining consent for the cochlear implant. You will have five minutes to pick your role (parent or physician) and have a full conversation including the benefits, risks, and alternatives.
   - 1. Benefits^2-4^
        - Aids in lip reading abilities and speech recognition alongside consistent auditory training
        - Increased perception of environment sounds (discrimination between loud, medium and soft sounds)
        - Aids in monitoring the volume of one’s own voice
     2. Risks
        - Surgical bleeding, infection, reaction to general anesthesia
        - Injury to the facial nerve (paralysis, taste changes)
        - Increased risk of meningitis
        - CSF or perilymph fluid leak
        - Vertigo, tinnitus, numbness around ear
        - Implant mechanical/electrical failure or rejection leading to a need for further surgeries
        - Loss of any residual hearing abilities in implanted ear
        - Variable results of implant: from normal ability to understand speech to no hearing benefit
     3. Alternatives
        - Use of some combination of lip reading, cued speech, and American Sign Language with an emphasis on early language acquisition
        - Use of hearing aids or assist devices in home/workspace
2. Please answer the following questions and discuss as a large group.
   1. What are the parameters involved in obtaining informed consent?
   2. Why is it important to offer interpreters and provide them when requested?
   3. What resources can you offer to these parents?

The Smiths engage in discussion, but voice opposition to the cochlear implant. They know the cochlear implant is not a quick or guaranteed fix, and that years of work would be needed for Ellie to learn to interpret the frequencies into spoken English. The Smiths explain that they do not see their deafness as a disability, and instead value their deafness as the source of their language, culture, and values. They plan to live with their child in the Deaf community, use ASL at home, and enroll Ellie in a school focused on this communication approach. They want to continue forward with the team of specialists for Ellie’s education, focusing on early language acquisition with ASL, but do not want the cochlear implant.

1. Please answer the following questions and discuss as a large group.
   1. Are the parents allowed to refuse a cochlear implant, even if it is medically advised to optimize Ellie’s chance at hearing?
   2. Are there any assumptions you made when advising the parents and how does this relate to the medical and social models of disability?
   3. How might this be different if the parents were hearing?
   4. What is shared decision-making and how does it relate to informed consent?

The information in this case has been simplified for educational purposes. With any community there is variation in opinions and there are many individuals with cochlear implants who consider themselves members of the Deaf community. If you’d like to learn more about the Deaf community, Deaf culture, or cochlear implants please see sources below.

<https://academic.oup.com/jdsde/article/11/1/102/410842>

<https://academic.oup.com/jdsde/article/15/2/162/551818>

<https://harmreductionjournal.biomedcentral.com/articles/10.1186/1477-7517-9-16>

<https://journals.sagepub.com/doi/full/10.1177/0142723719834102>

<https://www.ohio.edu/ethics/2001-conferences/cochlear-implants-the-deaf-culture-and-ethics/index.html>

<https://scholar.valpo.edu/cgi/viewcontent.cgi?article=1840&context=vulr>

<https://www.nad.org/resources/american-sign-language/community-and-culture-frequently-asked-questions/>

References

1. Mitchell RE KM. Chasing the mythical ten percent: Parental hearing status of deaf and hard of hearing students in the United States. *Sign Language Studies*. 2004;4(2):138-163.

2. *Benefits and Risks of Cochlear Implants*. 2021. *Medical Devices*. <https://www.fda.gov/medical-devices/cochlear-implants/benefits-and-risks-cochlear-implants>

3. *Cochlear Implant* 2021. *EVMS Ear Nose and Throat Surgeons*. <https://www.evms.edu/patient_care/specialties/ent_surgeons/services/otology/patient_education/cochlear_implant/>

4. *Cochlear Implantation, Iowa Head and Neck Protocols*. 2018. *Otolaryngology, Nursing, Speech Pathology, Patient Information*. <https://medicine.uiowa.edu/iowaprotocols/cochlear-implantation>
