# Supplementary material for: Shared Decision-Making at the Intersection of Disability, Culture, and Language Accessibility: An Educational Session for Medical Students
Source: MedEdPORTAL. 2024 Apr 30;20:11396. doi: 10.15766/mep_2374-8265.11396 (PMC11058081; doi:10.15766/mep_2374-8265.11396)
Supplement: Supplementary file 1 — Facilitator Guide.docxQuestions for Panelists.docxHearing and Listening.mp4Disability, Culture & Language Accessibility.pptxShared Decision-Making Lecture.mp4Session Guide.docxStudent Guide.docxSession Evaluation Tool.doc [file mep_2374-8265.11396-s001.zip › D. Disability, Culture & Language Accessibility.pptx]

## Slide 1
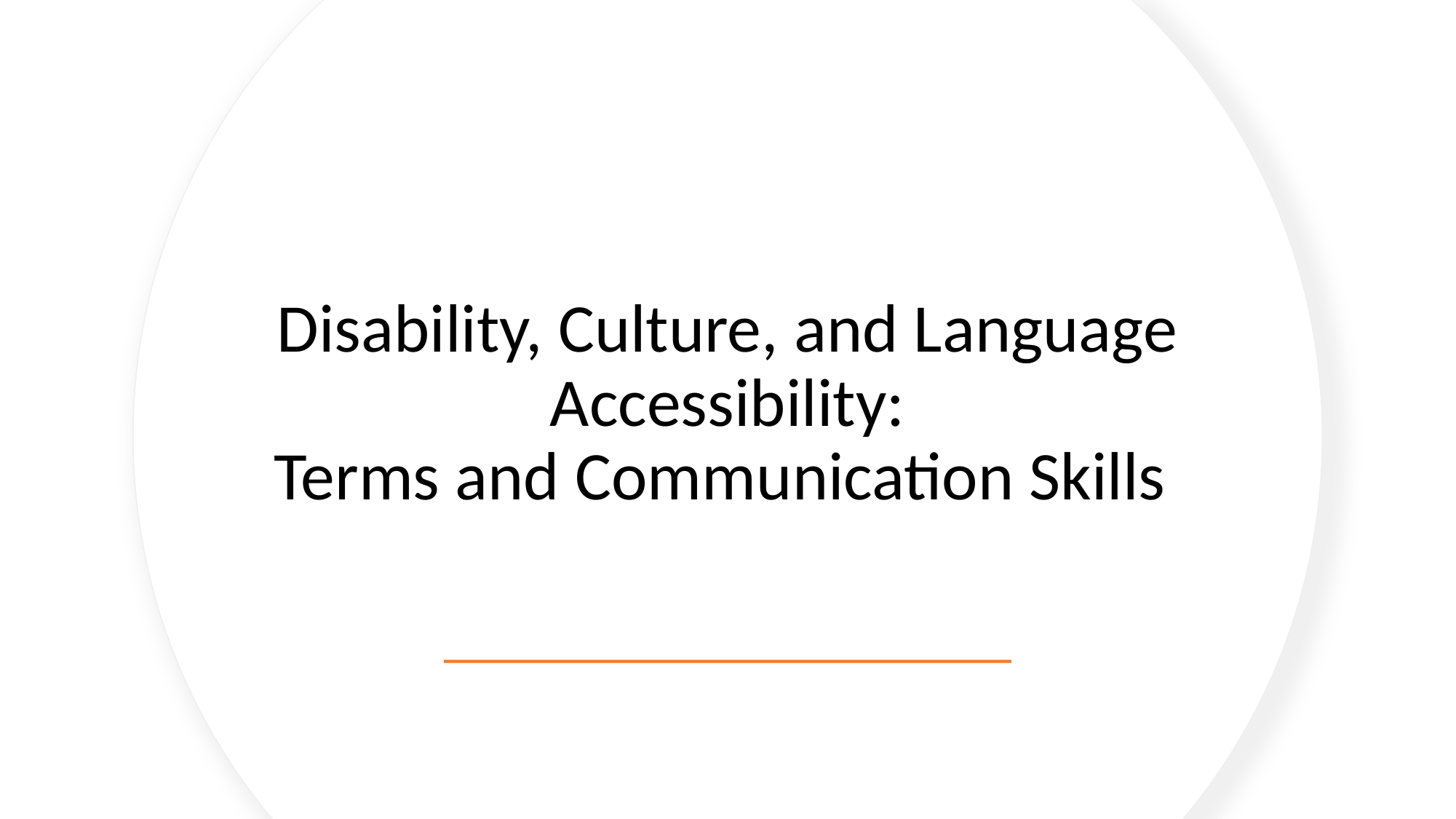

# Disability, Culture, and Language Accessibility: Terms and Communication Skills

## Slide 2
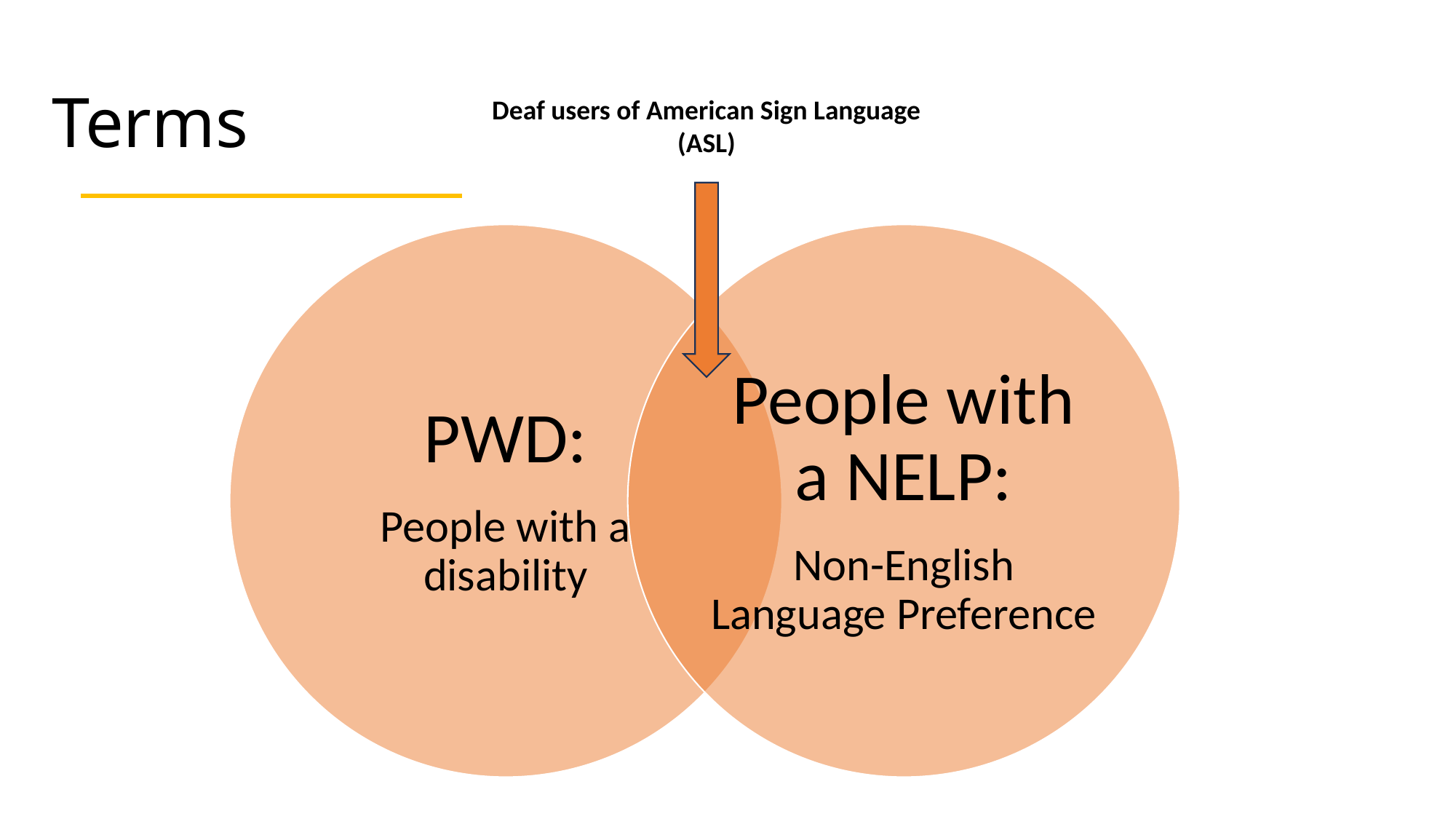

# Terms
Deaf users of American Sign Language (ASL)

## Slide 3
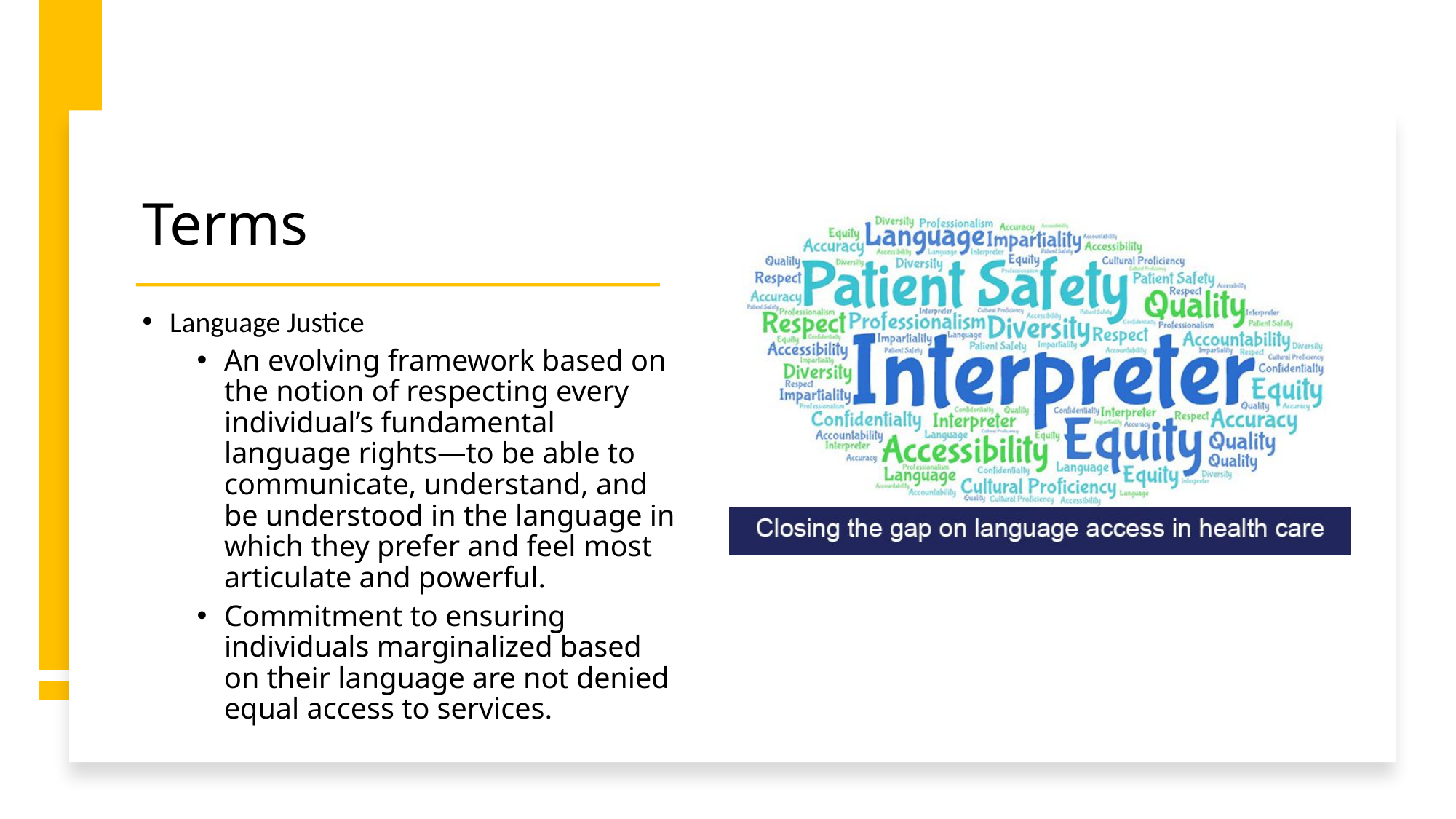

# Terms
Language Justice
An evolving framework based on the notion of respecting every individual’s fundamental language rights—to be able to communicate, understand, and be understood in the language in which they prefer and feel most articulate and powerful.
Commitment to ensuring individuals marginalized based on their language are not denied equal access to services.

## Slide 4
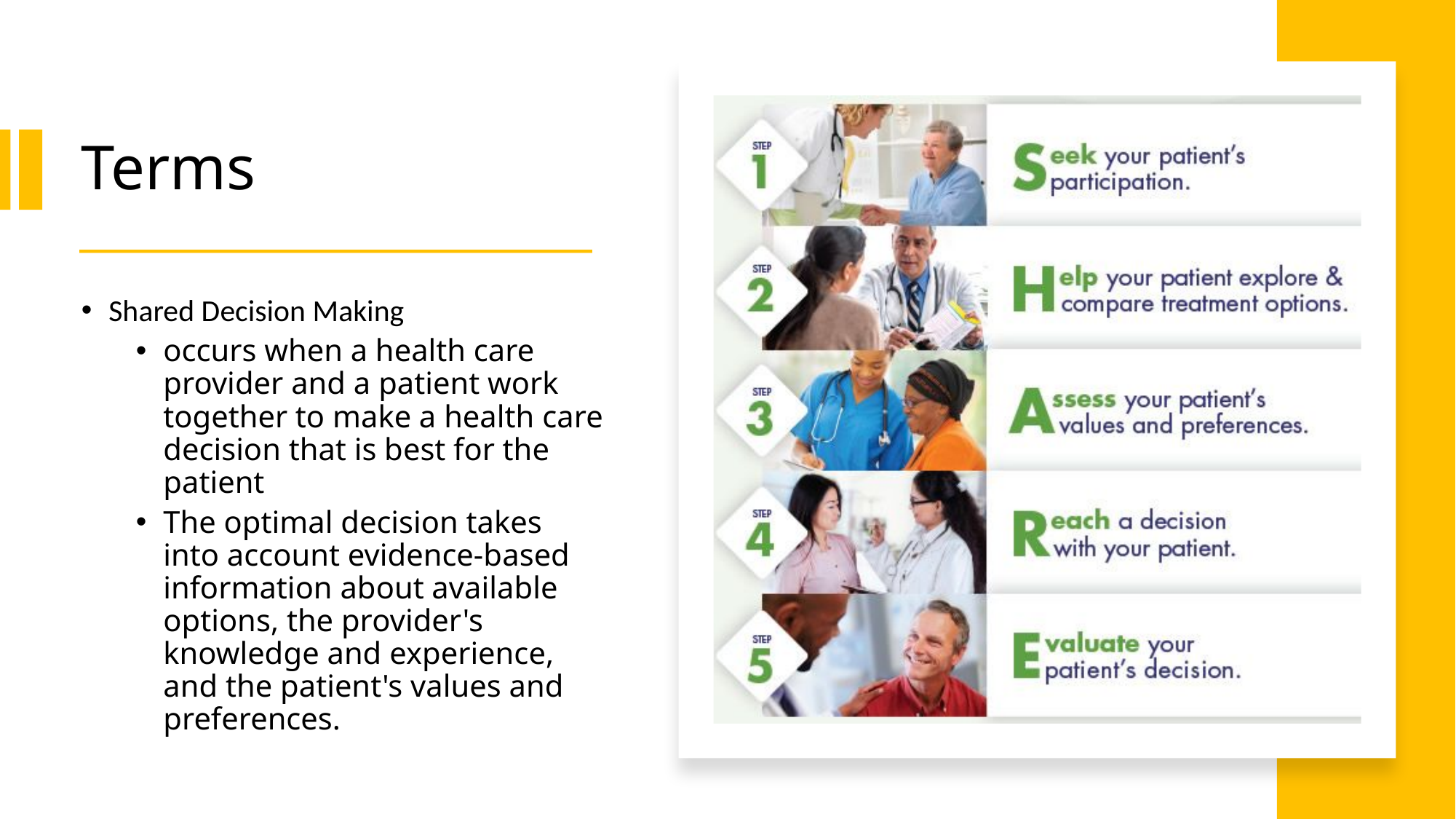

# Terms
Shared Decision Making
occurs when a health care provider and a patient work together to make a health care decision that is best for the patient
The optimal decision takes into account evidence-based information about available options, the provider's knowledge and experience, and the patient's values and preferences.

## Slide 5
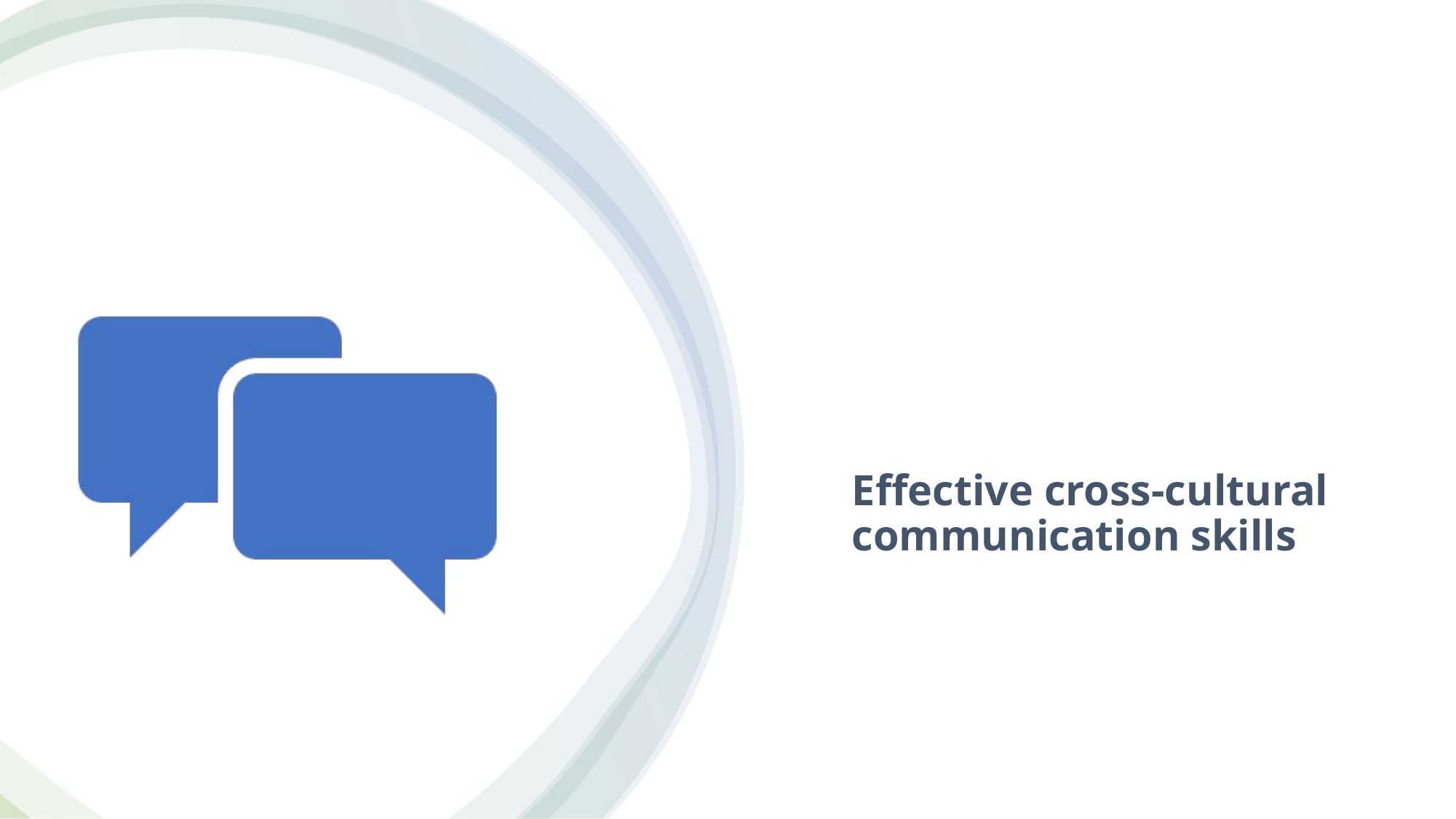

# Effective cross-cultural communication skills

## Slide 6
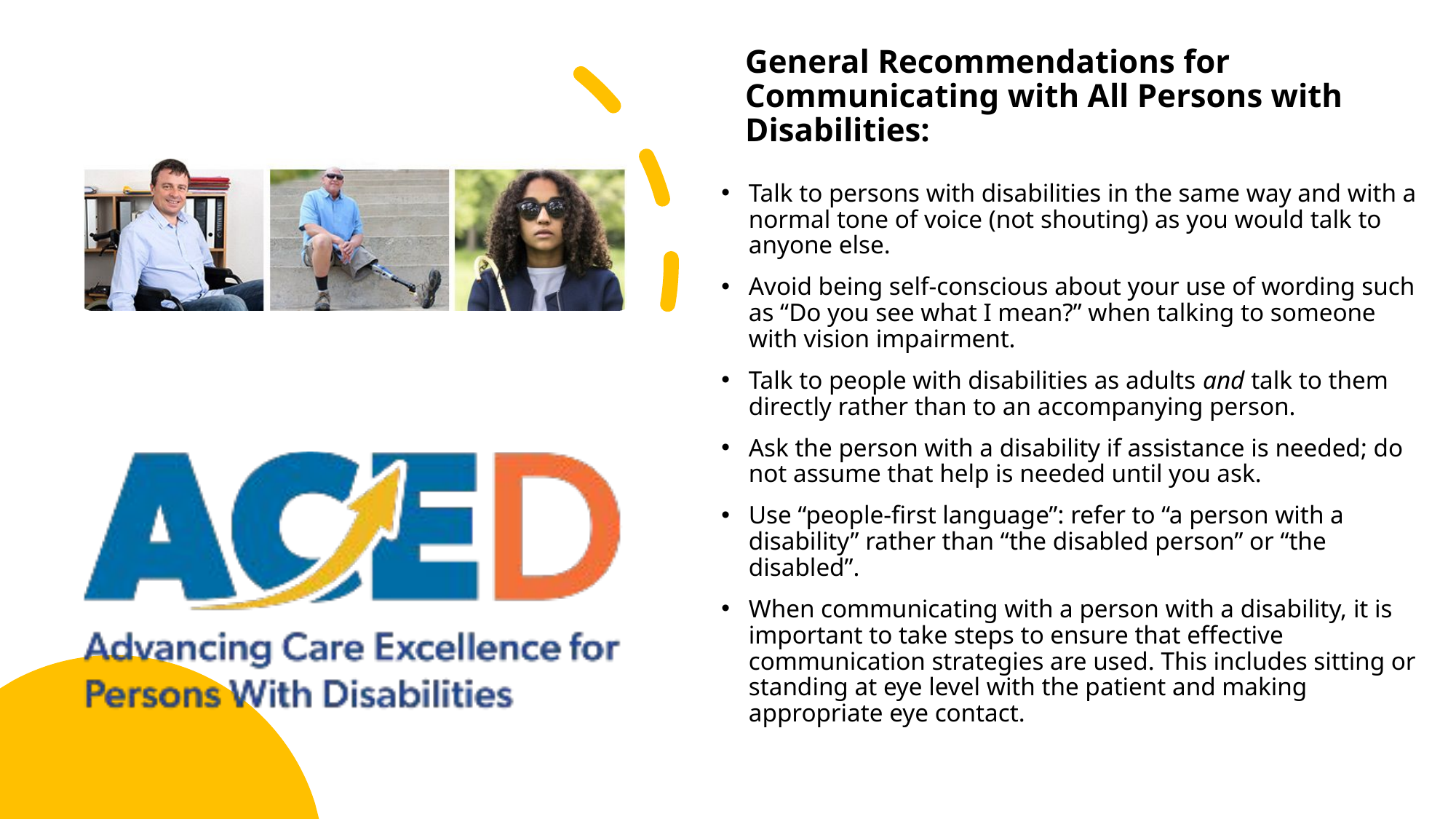

# General Recommendations for Communicating with All Persons with Disabilities:
Talk to persons with disabilities in the same way and with a normal tone of voice (not shouting) as you would talk to anyone else.
Avoid being self-conscious about your use of wording such as “Do you see what I mean?” when talking to someone with vision impairment.
Talk to people with disabilities as adults and talk to them directly rather than to an accompanying person.
Ask the person with a disability if assistance is needed; do not assume that help is needed until you ask.
Use “people-first language”: refer to “a person with a disability” rather than “the disabled person” or “the disabled”.
When communicating with a person with a disability, it is important to take steps to ensure that effective communication strategies are used. This includes sitting or standing at eye level with the patient and making appropriate eye contact.

## Slide 7
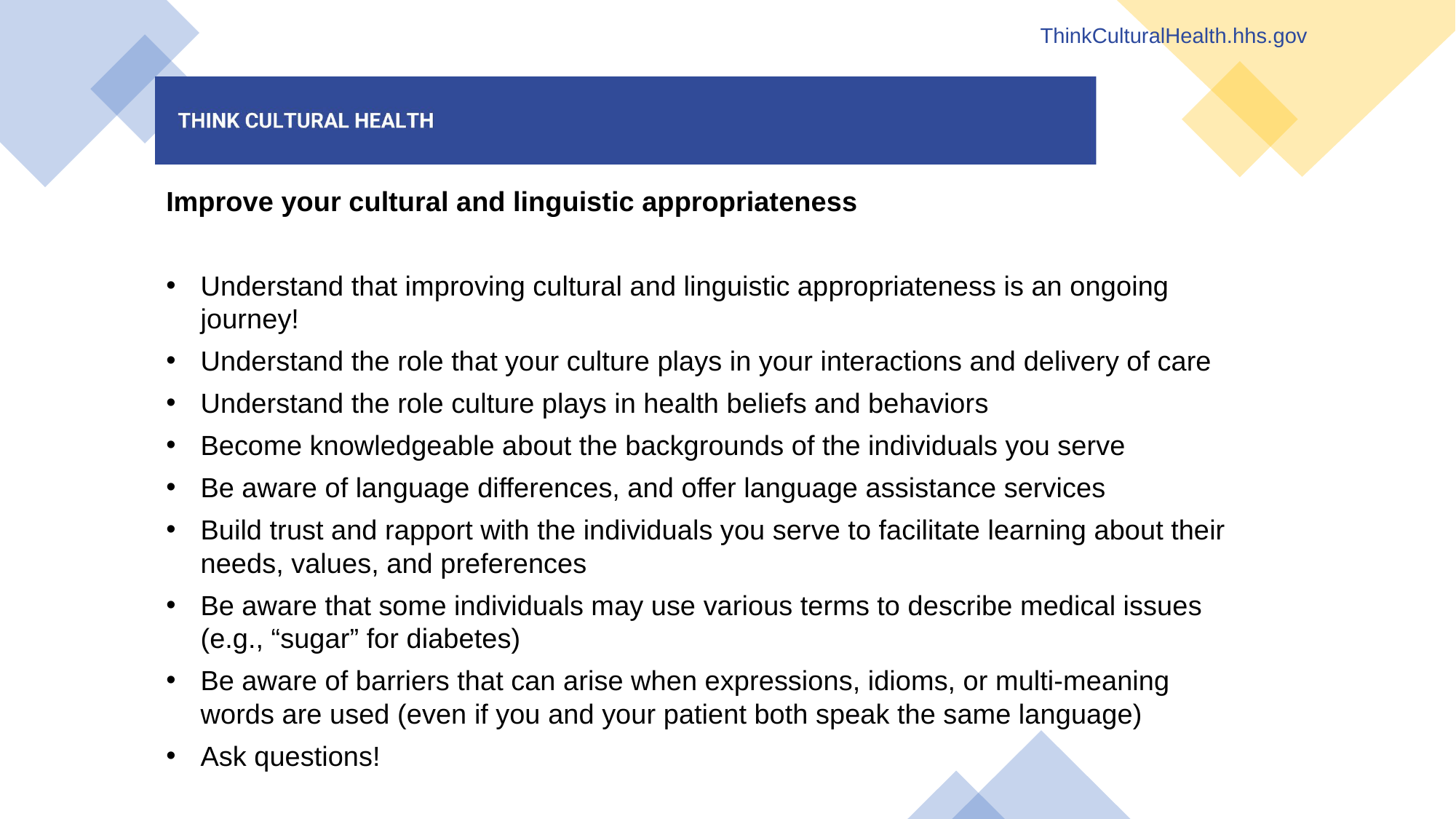

ThinkCulturalHealth.hhs.gov
Improve your cultural and linguistic appropriateness
Understand that improving cultural and linguistic appropriateness is an ongoing journey!
Understand the role that your culture plays in your interactions and delivery of care
Understand the role culture plays in health beliefs and behaviors
Become knowledgeable about the backgrounds of the individuals you serve
Be aware of language differences, and offer language assistance services
Build trust and rapport with the individuals you serve to facilitate learning about their needs, values, and preferences
Be aware that some individuals may use various terms to describe medical issues (e.g., “sugar” for diabetes)
Be aware of barriers that can arise when expressions, idioms, or multi-meaning words are used (even if you and your patient both speak the same language)
Ask questions!

## Slide 8
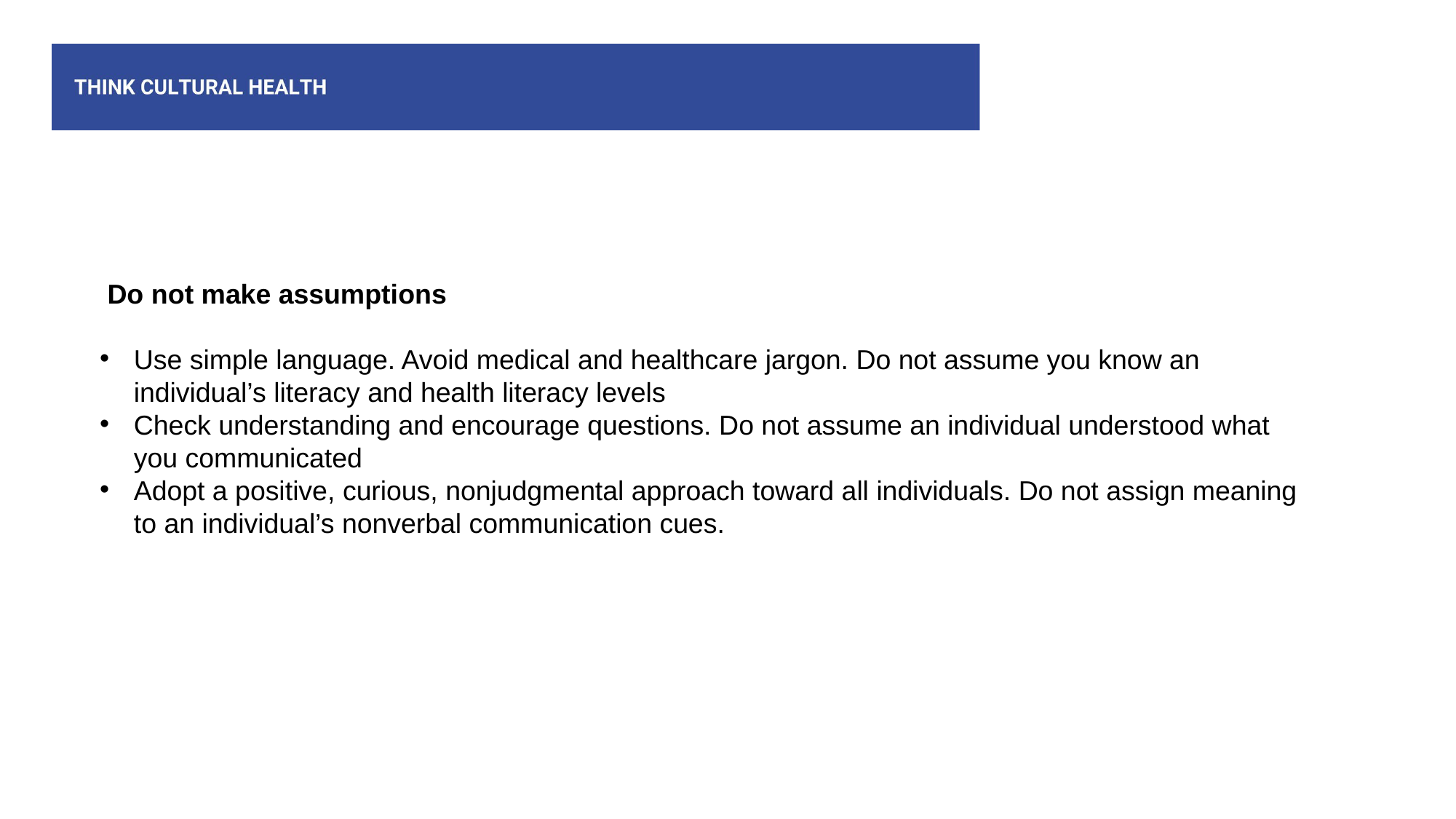

Do not make assumptions
Use simple language. Avoid medical and healthcare jargon. Do not assume you know an individual’s literacy and health literacy levels
Check understanding and encourage questions. Do not assume an individual understood what you communicated
Adopt a positive, curious, nonjudgmental approach toward all individuals. Do not assign meaning to an individual’s nonverbal communication cues.

## Slide 9
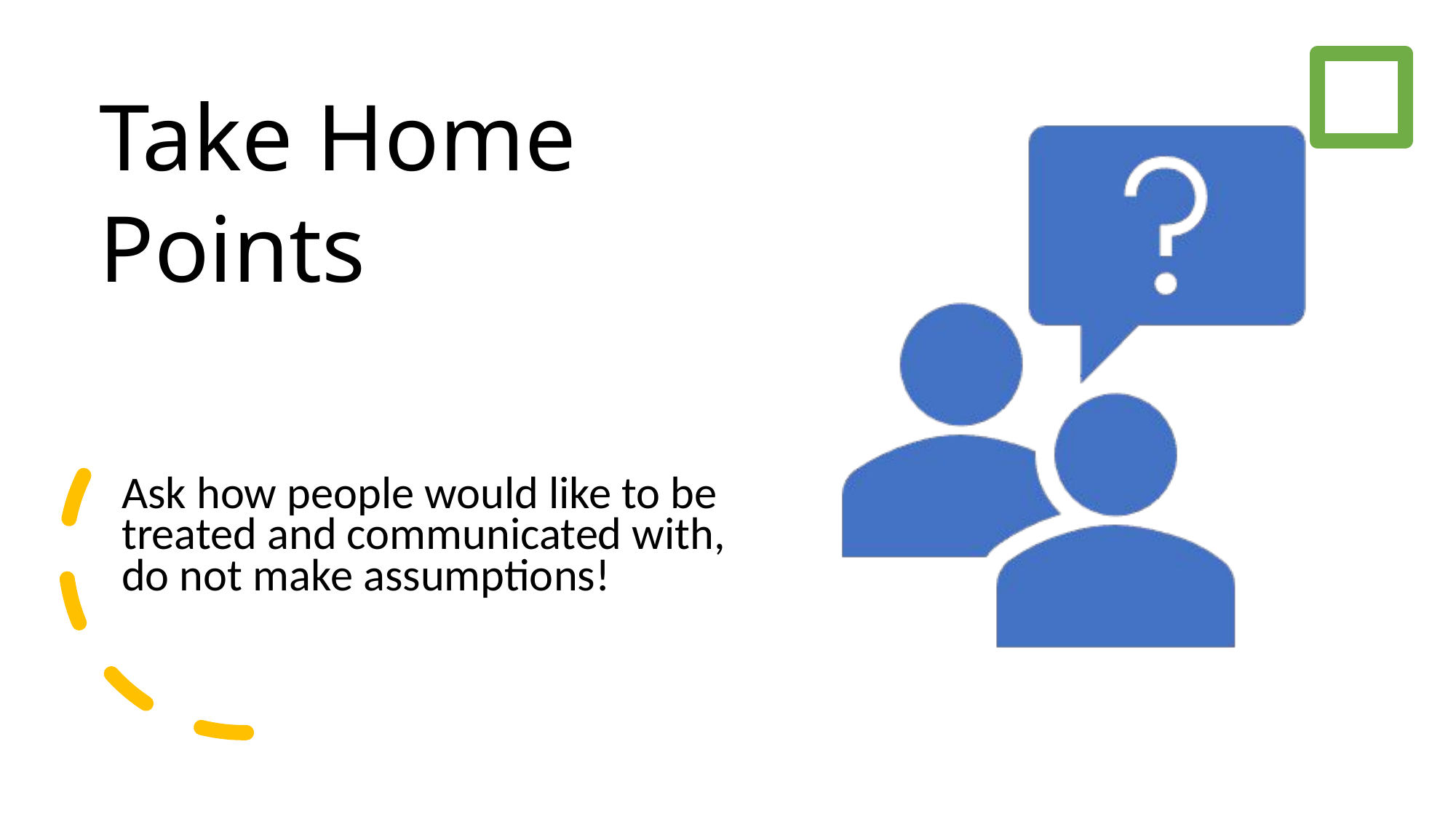

# Take Home Points
Ask how people would like to be treated and communicated with, do not make assumptions!
