# Supplementary material for: Shared Decision-Making at the Intersection of Disability, Culture, and Language Accessibility: An Educational Session for Medical Students
Source: MedEdPORTAL. 2024 Apr 30;20:11396. doi: 10.15766/mep_2374-8265.11396 (PMC11058081; doi:10.15766/mep_2374-8265.11396)
Supplement: Supplementary file 1 — Facilitator Guide.docxQuestions for Panelists.docxHearing and Listening.mp4Disability, Culture & Language Accessibility.pptxShared Decision-Making Lecture.mp4Session Guide.docxStudent Guide.docxSession Evaluation Tool.doc [file mep_2374-8265.11396-s001.zip › _Educational Summary Report_11396.pdf]

MedEdPORTAL® The AAMC Journal of Teaching and Learning Resources

Original Publication Open Access

Shared Decision-Making at the Intersection of Disability, Culture, and Language Accessibility: An Educational Session for Medical Students

Hannah Ship*, Sahana Shankar, Jeffrey P. Brosco, MD, PhD, Shelly Baer, LCSW, Sheryl Eisenberg Michalowski, MA, Jairo Arana, Damian Gregory, Ashley Falcon, PhD, MPH

*Corresponding author: his17@miami.edu

Abstract

Introduction: People with disabilities and those with non-English language preferences have worse health outcomes than their counterparts due to barriers to communication and poor continuity of care. As members of both groups, people who are Deaf users of American Sign Language have compounded health disparities. Provider discomfort with these specific demographics is a contributing factor, often stemming from insufficient training in medical programs. To help address these health disparities, we created a session on disability, language, and communication for undergraduate medical students. **Methods:** This 2-hour session was developed as a part of a 2020 curriculum shift for a total of 404 second-year medical student participants. We utilized a retrospective postsession survey to analyze learning objective achievement through a comparison of medians using the Wilcoxon signed rank test ($\alpha = .05$) for the first 2 years of course implementation. **Results:** When assessing 158 students' self-perceived abilities to perform each of the learning objectives, students reported significantly higher confidence after the session compared to their retrospective presession confidence for all four learning objectives ($ps < .001$, respectively). Responses signifying learning objective achievement (scores of 4, *probably yes,* or 5, *definitely yes*), when averaged across the first 2 years of implementation, increased from 73% before the session to 98% after the session. **Discussion:** Our evaluation suggests medical students could benefit from increased educational initiatives on disability culture and health disparities caused by barriers to communication, to strengthen cultural humility, the delivery of health care, and, ultimately, health equity.

Keywords
American Sign Language, Cultural Humility, Health Care Accessibility, Shared Decision-Making, Communication Skills, Cultural Competence, Disabilities, Diversity, Equity, Inclusion, Health Equity, Language-Appropriate Health Care

Educational Objectives

By the end of this activity, learners will be able to:

1. Identify that non-English language preference can act as a social determinant of health (e.g., in individuals who communicate using American Sign Language).
2. Apply elements of the medical and social models, practicing cultural humility, as appropriate in the context of Deaf culture.
3. Apply the key components of valid consent using a shared decision-making framework.
4. Describe the ability of all persons, regardless of disability, to provide valid consent that reflects respect for self-determination.

Citation:
Ship H, Shankar S, Brosco JP, et al. Shared decision-making at the intersection of disability, culture, and language accessibility: an educational session for medical students. *MedEdPORTAL.* 2024;20:11396. https://doi.org/10.15766/mep_2374-8265.11396

Introduction

Understanding and addressing health care disparities among vulnerable populations, specifically people with disabilities (PWD), stands as a critical endeavor in contemporary medical practice and research. The session presented here delves into the profound challenges faced by these marginalized communities, with a particular emphasis on those with sensory or cognitive conditions within the spectrum of PWD, people who are Deaf users of American Sign Language (ASL).[1] This group falls under both PWD and people with non-English language preferences (NELP). Both of these groups experience worse health outcomes than the general population, as ineffective interactions with providers and barriers to communication contribute to poor continuity of care.[2-5] As a result of insufficient medical trainee

Copyright © 2024 Ship et al. This is an open-access publication distributed under the terms of the Creative Commons Attribution license.

programs, data show that many providers are unsure how to effectively interact with PWD, NELP, and, as compounded, people who are Deaf users of ASL.[6,7]

It is rare that programs teach health providers about Deaf culture or ASL, which is surprising given that an estimated one to two million Americans belong to the Deaf community (capital D), one that is distinguished by the use of ASL and its unique culture.[8] Without an understanding of this community, as well as the intersection of cultural humility, language, and disability, health care professionals are unable to properly care for this population and address compounded health disparities.[9] Specifically in health care practices, this lack of communication and cultural understanding contributes to health disparities in the Deaf community.[1,10] When compared to the general public, patients who are Deaf users of ASL have been found to have fewer doctor's appointments, fewer preventative services, worse cardiovascular health outcomes, and higher rates of obesity.[11,12]

While the focus is primarily on people with sensory or cognitive conditions, it is imperative to acknowledge the emergence of the term *language justice,* recognizing the disparities stemming from NELP such as those who identify as Deaf users of ASL.[5] In fact, simply the presence of a physical communication barrier has been significantly associated with an increased risk of a preventable adverse event.[4] Studies have shown people with NELP consistently receive lower quality health care than English-proficient patients, evidenced by decreased understanding of treatment plans and disease processes, decreased postvisit satisfaction, and increased incidence of medical errors resulting in physical harm.[3] Moreover, the lack of effective health care communication leads to limitations in shared decision-making, hindering the collaborative process between practitioners and patients to arrive at health care decisions aligned with shared values.

Existing disability education models available in *MedEdPORTAL,* such as those by Borowsky, Morinis, and Garg,[13] Hearn and Hearn,[14] and Rogers, Morris, Hook, and Havyer,[15] adeptly introduce health care trainees to critical aspects of US disability policy, bias, and health care disparities linked to disability. Our module goes beyond this foundational knowledge by focusing on guiding learners to implement these concepts through the lens of cultural humility in health care communication. Recognizing the noticeable lack of emphasis on these crucial areas within current national medical education disability curricula, our module deliberately prioritizes tailored communication strategies specifically aimed at people who use ASL.[16] Furthermore, it fosters shared decision-making interactions between health care providers and patients with disabilities. This deliberate shift signifies a progressive step in disability education, acknowledging the pressing need for medical students to possess the competencies required to engage and support people with diverse needs while implementing frameworks that uphold cultural humility in medical practice.[17,18] In fact, research has shown that educational sessions focused on improving knowledge and skill sets in communication have a longer-lasting impact than changes in attitude.[19]

In contrast, Haugland and colleagues[20] and Dhanani and colleagues[21] have publications in *MedEdPORTAL* that identify communication barriers in health care for PWD and incorporate communication approaches using augmentative communication devices or strategies for interviewing patients on sensitive topics. While this is commendable, our educational session distinguishes itself by advancing the discourse specifically concerning the needs of people who are Deaf users of ASL and the implementation of shared decision-making processes that uphold cultural humility within the medical context. Historically, the University of Rochester School of Medicine and the University of California, San Diego, School of Medicine had learner modules engaging medical students with local high populations of people who were Deaf users of ASL, although these programs are currently inactive and difficult to employ without large, local, and active Deaf populations willing to volunteer.[22,23] There has not yet been an educational module that introduces people who are Deaf users of ASL and investigates the intersection of shared decision-making and disability cultural humility in patient-physician interactions. By addressing these distinct areas, our objective is to significantly enhance patient-centered care, thereby fostering a more inclusive and effective health care environment.

In our educational session, we spotlight people who are Deaf users of ASL. This approach allows for an exploration of various dimensions of cultural humility and of the patient-physician relationship, as well as addressing the needs of PWD, specifically those with sensory or cognitive conditions. By enhancing students' understanding of disability culture, promoting shared decision-making, and emphasizing the value of communication in health care, this program equips learners with the essential skills needed to interact effectively with diverse patient populations.

The session was created for second-year undergraduate medical students. These students, who started clinical rotations 4 months prior, have had direct interactions with patients in clinical and hospital settings. These experiences provided them with insights into current practices in patient-physician

Copyright © 2024 Ship et al. This is an open-access publication distributed under the terms of the Creative Commons Attribution license.

decision-making and language interpretation. During their first year, these students engaged in a small-group, case-based module exposing them to the fundamentals of valid consent. Additionally, they participated in two prior mandatory disability education sessions: Interdisciplinary Health Professions, emphasizing interdisciplinary teamwork and holistic patient care, and Disability Culture and Health Disparities, introducing concepts such as disability culture, implicit bias, health disparities, and the differentiation between medical and social models of disability. We strongly believe that our session, although designed as the third in this educational series, holds value for all health care workers, especially those directly involved in patient care, as it underscores the significance of effective health care communications.

Methods

Session Development and Context

The curriculum development team (CDT) developed this session as part of a larger curriculum shift at the University of Miami Miller School of Medicine into the NextGen Curriculum, in the Medicine as Profession (MAP) longitudinal course. The pilot class had 200 students, and the second-year class had 204 students. The CDT worked with course directors to implement this session during the year 2 curriculum, after students had completed at least one 12-week clinical rotation.

The CDT, consisting of PWD, medical students, legal advocates, and health care professionals, utilized Kern's six-step approach to curriculum development to develop the session and Bloom's taxonomy to create the learning objectives.[24,25] Facilitators for the small-group session were the longitudinal educational coordinators of the MAP course, faculty of the medical school who met with the students on a weekly basis for the MAP sessions. Prior to the session, we implemented a 30-minute faculty training session to orient them to the aims, themes, and structure of the session. We provided faculty with a facilitator guide for the small-group patient scenario and role-play exercise (Appendix A). We also held an orientation session for members of the optional large-group panel, consisting of PWD and their family members, introducing them to the panel questions and key themes of the session (Appendix B).

Our team created and curated one of the prework videos, Hearing and Listening: Health Equity, Deaf Culture, and Communication for Health Care Professionals, as a community needs assessment in advance of the session (Appendix C).[26] Funded by a University of Miami MD/MPH Population Health grant, the 7-minute video was designed to showcase the impact of language preferences on health as a social determinant and thereby support language justice in the field of medicine. The video featured real-life testimonials from people who were Deaf users of ASL, alongside insights from field experts and health and legal professionals. It delved into the barriers faced by those using ASL or who were Deaf or hard of hearing, addressing social determinants of health and limitations in achieving health equity. Students accessed the video on YouTube as a part of their prework material.

To build on the clinical implementation of the ideas introduced in the video, the CDT developed a resource for students in the form of a PowerPoint presentation entitled Disability, Culture, and Language Accessibility: Terms and Communication Skills (Appendix D). This presentation comprised a range of essential tools, encompassing definitions of key terms such as language justice and shared decision-making. It also offered valuable insights into effective cross-cultural communication skills in clinical settings. Moreover, the PowerPoint provided general recommendations for communicating with all persons with disabilities, developed by the Advancing Care Excellence for Persons With Disabilities program.[27] One of the key overarching messages highlighted throughout the presentation was the importance of inquiring about how individuals prefer to be treated and communicated with, emphasizing the avoidance of making assumptions. The primary focus was to equip students with a comprehensive understanding of essential ideas in the patient-physician relationship and effective communication skills in diverse cultural and disability contexts, ultimately emphasizing the significance of respectful and individualized communication practices.

The second prework video curated by the CDT included a faculty lecture by Jeffrey P. Brosco on shared decision-making entitled Family Professional Partnership: A Case Study in Communication (Appendix E). This video explained shared decision-making and a patient-physician relationship that would uphold the values and culture of the individual patient.

Session Content Outline

The phases of the session included time dedicated to student prework (3 hours), a small-group patient scenario discussion (45 minutes), and an optional panel with a large-group discussion (45 minutes).

1. The prework included two videos (Appendices C and E), one article, and one optional research article. Students were provided with the links directly, as well as with a session guide outlining the prework, the learning objectives, and the session structure (Appendix F). The

Copyright © 2024 Ship et al. This is an open-access publication distributed under the terms of the Creative Commons Attribution license.

required article was "Communication Considerations A-Z: Deaf Culture & Community,"[28] while the optional article was "Informed Consent."[29] In the second year of implementation, the "Communication Considerations A-Z: Deaf Culture & Community" article was removed due to student feedback about prework overload, and a self-navigational PowerPoint entitled Disability, Culture, and Language Accessibility: Terms and Communication Skills was added (Appendix D). Key themes of the prework included understanding Deaf culture from members of the community itself, shared decision-making reflecting the values of individuals, and how barriers to communication in health care could act as a social determinant of health.

2. The small-group sessions included a facilitated discussion on a patient scenario and student role-play exercise (Appendix G). The scenario described a clinical discussion with two parents identifying as Deaf and their decision on whether or not to obtain a cochlear implant for their child. Students were given the opportunity to discuss and apply themes of valid consent, shared decision-making, and disability/Deaf culture.
3. At our institution, the large-group panel followed the small groups on Zoom and featured four PWD and family members of PWD discussing their experiences communicating in health care settings. The panel was moderated by a PWD, who introduced the questions and allowed each panelist to respond (Appendix B). Panelists were encouraged to share both positive and negative experiences in health care. Key themes included disability culture, self-determination, and the importance of accessible, open communication.
4. After the session, students were supplied with the patient scenario and role-play exercise facilitator guide with resources for further learning (Appendix A). Students also received the link for the evaluation survey (Appendix H).

Optional Panel

We highly recommend the inclusion of the optional large-group panel, as it offers students an invaluable opportunity to directly interact with PWD. While recognizing that access to panelists might vary among programs and universities and that this experience cannot be universally standardized, we have aimed to support the panel's implementation. To facilitate this, we have included a document containing panelist questions and broad themes for a 30-minute orientation discussion (Appendix B). Furthermore, the associated learning objective 4, which focuses on describing the ability of all individuals, regardless of disability, to provide valid consent while respecting their self-determination, is concurrently addressed through the patient scenario discussion and role-play exercise.

Evaluation Strategy

After the session, students received a survey to retrospectively evaluate execution of the learning objectives and the session's educational quality (Appendix H). We utilized the retrospective pre- and postquestionnaire design originally created by Donald Campbell and Julian Stanley in 1963.[30] This approach was adopted with the objectives of mitigating response shift and strengthening the precision of measuring of change.[31] Response shift bias manifests when a participant uses a different frame of understanding about a question between the pre and post periods. The survey was created on Qualtrics, an electronic survey software, and was provided to the students in the session materials, by email, and on the class's GroupMe communication page. The main survey questions utilized a 5-point Likert scale (1 = *definitely no*, 2 = *probably no*, 3 = *might or might not*, 4 = *probably yes*, 5 = *definitely yes*) to assess students' self-perceived abilities to perform each of the learning objectives. The survey also included questions about the quality of learning of each segment (prework materials, small-group patient scenario and role-play exercise, and large-group panel) and two free-response questions, allowing students to discuss what they would have liked more and less time with. The survey was anonymous and did not collect participants' identifiable information. The analysis included comparisons of median pre- and postsurvey data from all participants using the Wilcoxon signed rank test ($\alpha = .05$) and effect size calculation of the analytical sample.

Results

The pilot year class (class of 2024) had 200 students with a response rate of 55% (109), and the second-year class (class of 2025) had 204 students with a response rate of 24% (49). Between both classes, this was an overall response rate of 39% (158 out of 404). Demographics were not collected as this survey was an anonymous one given to the entire class.

For both years of implementation, when evaluating students' self-perceived abilities to perform each of the learning objectives (confidence), students reported significantly higher confidence after the session as compared to their retrospective presession confidence for all four of the learning objectives (Table). This was found to be significant, with a large effect size for all learning objectives ($p < .05$, effect size > 0.50).

For the pilot year, 98% of responses signified achievement of the learning objectives (scores of 4, *probably yes,* and 5,

Copyright © 2024 Ship et al. This is an open-access publication distributed under the terms of the Creative Commons Attribution license.

Table. Learners' Perception of Addressing the Learning Objectives Pre- and Postsession

Learning Objective[a,b]		Class	Presession *Mdn* (IQR)[c]	Postsession *Mdn* (IQR)	*N*	*p*
Pair 1	Recognize that limited English proficiency is a social determinant of health (for example, in individuals who communicate using American Sign Language).	2024	4 (1)	5 (0)	109	<.01
		2025	4 (1)	5 (0)	49	<.01
		Combined	4 (1)	5 (0)	158	<.01
Pair 2	Apply elements of the medical and social models as appropriate in the context of Deaf culture.	2024	3 (1)	5 (1)	107	<.01
		2025	4 (1)	5 (1)	48	<.01
		Combined	3 (1)	5 (1)	155	<.01
Pair 3	Apply the key components of valid consent using a shared decision-making framework.	2024	4 (0)	5 (1)	109	<.01
		2025	4 (1)	5 (1)	49	<.01
		Combined	4 (0)	5 (1)	158	<.01
Pair 4	Value the ability of all persons, regardless of disability, to provide valid consent that reflects respect for self-determination.	2024	4 (1)	5 (0)	109	<.01
		2025	5 (1)	5 (0)	49	<.01
		Combined	4 (1)	5 (0)	158	<.01

Abbreviation: IQR, interquartile range.
[a]Rated on a 5-point Likert scale (1 = *definitely no*, 2 = *probably no*, 3 = *might or might not*, 4 = *probably yes*, 5 = *definitely yes*).
[b]Objectives' language has been updated in the main text for future iterations; the language here is specific to the implementations with the classes of 2024 and 2025.
[c]Presession data were obtained in a retrospective survey, concurrently with postsession data. The analysis included comparisons of median pre- and postsurvey data from all participants using the Wilcoxon signed rank test ($p < .05$).

definitely yes) after the session. This was in contrast to only 72% of the responses signifying achievement before the session (Figure). These values were similar to the second-year class implementation, with 99% of responses signifying achievement after the session, compared to 74% before the session.

Questions 2 and 3 on the survey aimed to assess the educational quality of the center-based activities and prework, respectively. Combining the data from both years ($n = 175$), the prework received a median score of 3 (interquartile range [IQR] = 1), the patient scenario and role-play exercise received a median score of 4 (IQR = 1), and the family and patient panel received a median score of 4 (IQR = 1). When specifically rating the educational quality of the prework, the Hearing and Listening video received a median score of 4 (IQR = 1), and the "Communication Considerations A-Z: Deaf Culture & Community" article received a median score of 3 (IQR = 1).

Themes of open-ended qualitative feedback across both years of implementation included desire to learn more ASL and enjoying learning ASL in the prework video. Responses included "I also really loved the Hearing and Listening video, especially the part towards the end where we learned a few important words in ASL that we can use when interviewing patients" and "It would have been nice to learn even more important signs (Do you need help? Can you read lips? etc.)." A few students did report prework fatigue and said that they had not read the articles. Many student responses also centered on the panel, including "The panel is truly fantastic! Highlight of our med school education" and "I really enjoyed the panel and the videos. I think it's important for people to understand and listen to peoples' experiences, especially in the context of intrapersonal reflection and growth."

Discussion

This is one of the first disability education activities mandated within an undergraduate medical curriculum that integrates communication and shared decision-making as fundamental themes in understanding disability culture and the nuanced intersection of language.[16] This type of medical education can have a widespread impact on minority groups, including PWD and those with NELP. These patient populations exist in every setting, and therefore, this training is necessary for and valuable to all health care professionals. With provider cultural humility acting as a social determinant of health, including this session is more important than ever—and tying it to standard medical education can help solidify its inclusion.

In reflecting on development, our team considered inclusivity of PWD and of lived experiences as a formidable strength throughout the development and implementation phases. We aligned our curriculum development with the disability rights movement slogan "Nothing about us without us." We found that including PWD in our CDT and on the large-group panel was a strength of the session. Our findings indicate that students highly valued the direct interactions with PWD through the optional large-group panel. This is supported by current research, which indicates that a disability curriculum is most impactful when including direct interaction with PWD.[15] To implement the session elsewhere, it would be helpful for a site to have access to its own experts in disabilities, to families and self-advocates in the area, and to people who identify with Deaf culture. A notable illustration of this approach is the integration of a community needs assessment in the form of the Hearing and Listening video included in the prework materials (Appendix C). This

Copyright © 2024 Ship et al. This is an open-access publication distributed under the terms of the Creative Commons Attribution license.

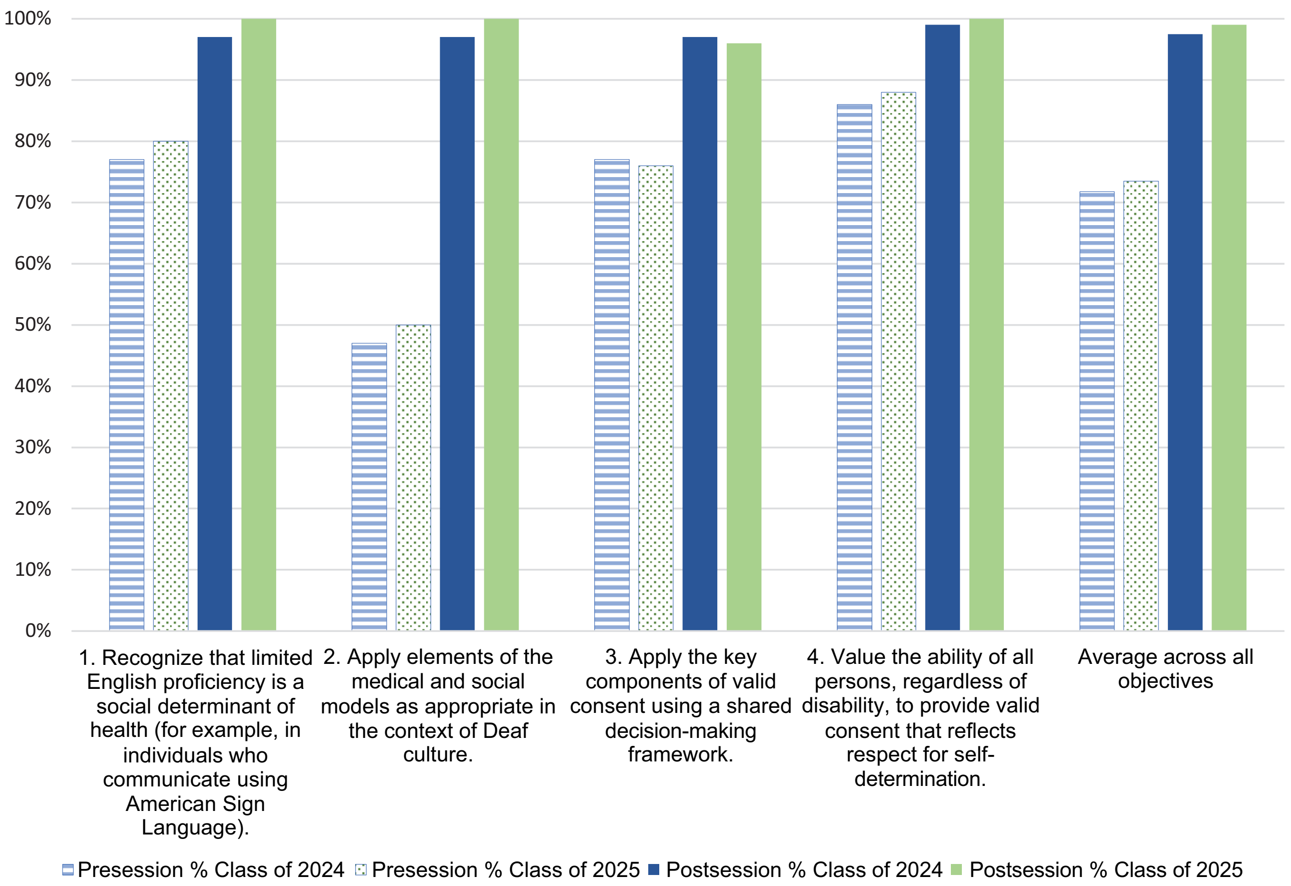

Figure. Learners' perception of addressing the learning objectives pre- and postsession. Scores of 4, *probably yes,* and 5, *definitely yes,* on a 5-point Likert scale signify achievement of the learning objectives. Objectives' language has since been updated for future iteration; the phrasing here was specific to the implementations with the classes of 2024 and 2025.

deliberate choice provided students with direct insights into the firsthand experiences of members of the Deaf community and Deaf law advocates, vividly showcasing the profound impact of communication barriers on health equity within this community. Furthermore, the community needs assessment was able to act not only as an exploration of the problem in the community but also as an educational exposure for students. The duality in the resource allowed us to support the need for this instruction on an institutional level and also on a learner level. Lastly, a crucial lesson from our session design and implementation was linking social medicine ideals with traditional medical education as a technique to soften institutional buy-in and ease of incorporation. From this perspective, an added value of the patient scenario and role-play exercise (Appendix A) is that students can apply their foundational education in informed consent and interpreter use.

After evaluation, our team utilized the data in order to improve this session. Based on the feedback asking for more specialized instruction on ASL and community connection, we organized optional ASL workshops for students via collaboration with the University of Miami Miller School of Medicine Debbie Project organization. We are now organizing an ASL track through the University of Miami Disability Health Alliance to allow students to further connect with the local community and people who are hearing impaired. Based on the feedback in the first implementation requesting less prework, we removed the "Communication Considerations A-Z: Deaf Culture & Community" article from the prework. The Disability, Culture, and Language Accessibility prework PowerPoint (Appendix F) was added in response to feedback to support learners in organizing the terminology of the session and as a source of clinical skills when interacting with PWD. Lastly, the learning objectives' language was slightly altered in 2023 to be more specific and measurable for accuracy of assessment. The Table and Figure maintain the objectives' previous language to accurately represent learners' survey experience.

Copyright © 2024 Ship et al. This is an open-access publication distributed under the terms of the Creative Commons Attribution license.

Our inclusion of people who are Deaf users of ASL introduced a vital perspective from the disability community. This perspective underscored the importance of cultural humility, language justice, and understanding the unique needs of both people with NELP and PWD. Other studies have shown that increased comfort with these groups leads to better clinical interactions and decreased health disparities.[18] Based on our findings, students benefited from learning not just about disability culture but also about how cultural humility functions in patient interactions. As demonstrated by the significantly increased reported confidence with the fourth learning objective, the educational session was impactful in conveying the key themes of valuing all people and respecting patient self-determination.

We recognize the limitations in the evaluation method, including the potential for desirability bias as students' self-report their measure of achievement, though survey anonymity may have reduced the likelihood for this type of bias. Furthermore, medical students often have limited time and survey fatigue, which could lead to a low response rate, as we recognized specifically in the second year of implementation. We also recognize the use of retrospective measurement of baseline measures as a potential threat to internal validity. As also described by Hearn and Hearn,[14] we found that the retrospective pre-post questionnaire offers advantages in decreasing assumption bias and requiring students to indicate how much their confidence level has changed from before the session as an effect of the session itself. This avoids a ceiling effect in which students overestimate their baseline abilities before being exposed to the material of the session and data are not able to detect a change from students' self-perceived high scores. This retrospective pre-post questionnaire has the added benefit of reducing time burden and survey fatigue among medical students.

Expanding upon our evaluation findings, it becomes evident that enhancing medical students' exposure to disability culture and addressing health disparities stemming from communication barriers could significantly strengthen cultural humility, health care delivery, and overall health equity. Moving forward, our efforts will pivot towards forging stronger community partnerships between medical education and community organizations to reflect educational standards that represent the population's culture and needs. Additionally, we aspire to conduct more comprehensive and longitudinal evaluations to delve deeper into the session's educational effects and gauge its long-term influence on health care practices. The implications of our work transcend mere institutional boundaries, advocating for the fundamental integration of cultural humility and language accessibility within medical education policies. Empowered by diverse perspectives, future practitioners can aspire to dismantle health care disparities, champion equitable care, and empower every individual they serve. This journey towards inclusivity and equitable health care is imperative in shaping a compassionate and responsive health care landscape.

Appendices

A. Facilitator Guide.docx
B. Questions for Panelists.docx
C. Hearing and Listening.mp4
D. Disability, Culture & Language Accessibility.pptx
E. Shared Decision-Making Lecture.mp4
F. Session Guide.docx
G. Student Guide.docx
H. Session Evaluation Tool.doc

All appendices are peer reviewed as integral parts of the Original Publication.

Hannah Ship: Third-Year Medical Student, University of Miami Miller School of Medicine; ORCID: https://orcid.org/0000-0003-3031-9197

Sahana Shankar: Fourth-Year Medical Student, University of Miami Miller School of Medicine

Jeffrey P. Brosco, MD, PhD: Professor, Department of Pediatrics, University of Miami Miller School of Medicine

Shelly Baer, LCSW: Licensed Clinical Social Worker, Mailman Center for Child Development, University of Miami Miller School of Medicine

Sheryl Eisenberg Michalowski, MA: Deaf Advocate, Eisenberg & Baum Law Center for Deaf and Hard of Hearing

Jairo Arana: Clinical Program Coordinator, Mailman Center for Child Development, University of Miami Miller School of Medicine

Damian Gregory: Consultant, Mailman Center for Child Development, University of Miami Miller School of Medicine

Ashley Falcon, PhD, MPH: Associate Professor, School of Nursing and Health Sciences, University of Miami

Disclosures
None to report.

Funding/Support
None to report.

Prior Presentations
Ship H, Shankar S, Baer R, et al. Educational initiatives on disability in undergraduate medical education. Paper presented virtually at:

Copyright © 2024 Ship et al. This is an open-access publication distributed under the terms of the Creative Commons Attribution license.

2022 Alliance for Disability in Health Care Education (ADHCE) Annual Conference Meeting; June 9, 2022.

Shankar S, Ship H, Falcon A, et al. Disability culture, health disparities, and communication: a two-part disability curriculum for undergraduate medical students. Paper presented at: Society for Developmental & Behavioral Pediatrics Annual Meeting; October 21, 2022; Denver, CO.

Ship H, Shankar S, Brosco J, et al. Shared decision making in the setting of disability culture and limited English proficiency: an undergraduate medical school curriculum. Poster presented virtually at: Society for Medical Decision Making Annual North American Meeting; October 23-26, 2022.

Ship H, Shankar S, Brosco J, et al. Disability culture and communication in healthcare settings: a novel undergraduate medical school curriculum. Paper presented at: Association of University Centers on Disabilities (AUCD) Annual Meeting; November 16, 2022; Washington, DC.

Ship H, Shankar S, Brosco J, et al. Disability culture and communication in healthcare settings: a novel undergraduate medical school curriculum. Poster presented virtually at: American Academy of Developmental Medicine & Dentistry (AADMD) Meeting; May 5, 2023.

Ethical Approval

Reported as not applicable.

References

1. Schoenborn CA, Heyman K. *Health Disparities Among Adults With Hearing Loss: United States, 2000-2006*. Centers for Disease Control and Prevention; 2008. Accessed March 20, 2024. https://www.cdc.gov/nchs/data/hestat/hearing00-06/hearing00-06.pdf
2. Krahn GL, Walker DK, Correa-De-Araujo R. Persons with disabilities as an unrecognized health disparity population. *Am J Public Health*. 2015;105(suppl 2):S198-S206. https://doi.org/10.2105/AJPH.2014.302182
3. Green AR, Nze C. Language-based inequity in health care: who is the "poor historian"? *AMA J Ethics*. 2017;19(3):263-271. https://doi.org/10.1001/journalofethics.2017.19.3.medu1-1703
4. Bartlett G, Blais R, Tamblyn R, Clermont RJ, MacGibbon B. Impact of patient communication problems on the risk of preventable adverse events in acute care settings. *CMAJ*. 2008;178(12): 1555-1562. https://doi.org/10.1503/cmaj.070690
5. Quadri NS, Wilkins S, Krohn K, Mann EM, Stauffer WM, Walker PF. Language justice: addressing linguistic disparities begins with language data collection. *Am J Trop Med Hyg*. 2023;109(1):1-3. https://doi.org/10.4269/ajtmh.23-0237
6. Barnett S. Cross-cultural communication with patients who use American Sign Language. *Fam Med*. 2002;34(5):376-382.
7. Iezzoni LI, Rao SR, Ressalam J, et al. Physicians' perceptions of people with disability and their health care. *Health Aff (Millwood)*. 2021;40(2):297-306. https://doi.org/10.1377/hlthaff.2020.01452
8. Steinberg AG, Barnett S, Meador HE, Wiggins EA, Zazove P. Health care system accessibility: experiences and perceptions of deaf people. *J Gen Intern Med*. 2006;21(3):260-266. https://doi.org/10.1111/j.1525-1497.2006.00340.x
9. Tamaskar P, Malia T, Stern C, Gorenflo D, Meador H, Zazove P. Preventive attitudes and beliefs of deaf and hard-of-hearing individuals. *Arch Fam Med*. 2000;9(6):518-525. https://doi.org/10.1001/archfami.9.6.518
10. Kuenburg A, Fellinger P, Fellinger J. Health care access among deaf people. *J Deaf Stud Deaf Educ*. 2016;21(1):1-10. https://doi.org/10.1093/deafed/env042
11. Barnett S, Franks P. Health care utilization and adults who are deaf: relationship with age at onset of deafness. *Health Serv Res*. 2002;37(1):103-118. https://doi.org/10.1111/1475-6773.99106
12. Barnett S, Klein JD, Pollard RQ Jr, et al. Community participatory research with deaf sign language users to identify health inequities. *Am J Public Health*. 2011;101(12):2235-2238. https://doi.org/10.2105/AJPH.2011.300247
13. Borowsky H, Morinis L, Garg M. Disability and ableism in medicine: a curriculum for medical students. *MedEdPORTAL*. 2021;17:11073. https://doi.org/10.15766/mep_2374-8265.11073
14. Hearn SL, Hearn PJ. Working with people with disabilities: an interactive video/lecture session for first- and second-year medical students. *MedEdPORTAL*. 2020;16:10913. https://doi.org/10.15766/mep_2374-8265.10913
15. Rogers JM, Morris MA, Hook CC, Havyer RD. Introduction to disability and health for preclinical medical students: didactic and disability panel discussion. *MedEdPORTAL*. 2016;12:10429. https://doi.org/10.15766/mep_2374-8265.10429
16. Ratakonda S, Argersinger DP, Auchus GC, et al. A call for disability health curricula in medical schools. *Trends Mol Med*. 2022;28(12):1012-1015. https://doi.org/10.1016/j.molmed.2022.08.004
17. Ioerger M, Flanders RM, French-Lawyer JR, Turk MA. Interventions to teach medical students about disability: a systematic search and review. *Am J Phys Med Rehabil*. 2019; 98(7):577-599. https://doi.org/10.1097/PHM.0000000000001154
18. Sharby N, Martire K, Iversen MD. Decreasing health disparities for people with disabilities through improved communication strategies and awareness. *Int J Environ Res Public Health*. 2015; 12(3):3301-3316. https://doi.org/10.3390/ijerph120303301
19. Gutiérrez-Puertas L, Márquez-Hernández VV, Gutiérrez-Puertas V, Granados-Gámez G, Aguilera-Manrique G. Educational interventions for nursing students to develop communication skills with patients: a systematic review. *Int J Environ Res Public Health*. 2020;17(7):2241. https://doi.org/10.3390/ijerph17072241
20. Haugland M, Hartmann K, Feinn R, Gowdy L, Marquis-Eydman T. Interprofessional approach to educate health care students about

Copyright © 2024 Ship et al. This is an open-access publication distributed under the terms of the Creative Commons Attribution license.

intellectual and developmental disabilities: adaptive communication and physical activity planning. *MedEdPORTAL*. 2023;19:11317. https://doi.org/10.15766/mep_2374-8265.11317

21. Dhanani Z, Huynh N, Tan L, Kottakota H, Lee R, Poullos P. Deconstructing ableism in health care settings through case-based learning. *MedEdPORTAL*. 2022;18:11253. https://doi.org/10.15766/mep_2374-8265.11253
22. Mathews JL, Parkhill AL, Schlehofer DA, Starr MJ, Barnett S. Role-reversal exercise with Deaf Strong Hospital to teach communication competency and cultural awareness. *Am J Pharm Educ*. 2011;75(3):53. https://doi.org/10.5688/ajpe75353
23. Hoang L, LaHousse SF, Nakaji MC, Sadler GR. Assessing deaf cultural competency of physicians and medical students. *J Cancer Educ*. 2011;26(1):175-182. https://doi.org/10.1007/s13187-010-0144-4
24. Kern DE, Thomas PA, Howard DM, Bass EB. *Curriculum Development for Medical Education: A Six-Step Approach*. Johns Hopkins University Press; 1998.
25. Chatterjee D, Corral J. How to write well-defined learning objectives. *J Educ Perioper Med*. 2017;19(4):E610. https://doi.org/10.46374/volxix-issue4-chatterjee
26. Ship H. Hearing and listening: health equity, Deaf culture, and communication for healthcare professionals. YouTube. November 29, 2022. Accessed March 20, 2024. https://youtu.be/TD5VrsaEWMw
27. Smeltzer SC, Mariani B, Meakim C. Communicating with people with disabilities. Advancing Care Excellence for Persons With Disabilities. January 28, 2017. Updated December 5, 2023. Accessed March 20, 2024. https://www.nln.org/education/teaching-resources/professional-development-programsteaching-resourcesace-all/ace-d/additional-resources/communicating-with-people-with-disabilities-e030c45c-7836-6c70-9642-ff00005f0421
28. Benedict BS, Legg J. Communication considerations A-Z: Deaf culture & community. Hands & Voices. Accessed March 20, 2024. https://www.handsandvoices.org/comcon/articles/pdfs/deafculture.pdf
29. McCullough LB, Chervenak FA. Informed consent. *Clin Perinatol*. 2007;34(2):275-285. https://doi.org/10.1016/j.clp.2007.03.005
30. Campbell DT, Stanley JC. *Experimental and Quasi-Experimental Designs for Research.* Houghton Mifflin; 1963.
31. Synthesis of literature relative to the retrospective pretest design. AEA Connect: Teaching of Evaluation. October 29, 2005. Updated April 14, 2010. Accessed March 20, 2024. https://comm.eval.org/teaching/viewdocument/synthesis-of-literat

Received: July 24, 2023
Accepted: January 12, 2024
Published: April 30, 2024

Copyright © 2024 Ship et al. This is an open-access publication distributed under the terms of the Creative Commons Attribution license.
