# Supplementary material for: Shared Decision-Making at the Intersection of Disability, Culture, and Language Accessibility: An Educational Session for Medical Students
Source: MedEdPORTAL. 2024 Apr 30;20:11396. doi: 10.15766/mep_2374-8265.11396 (PMC11058081; doi:10.15766/mep_2374-8265.11396)
Supplement: Supplementary file 1 — Facilitator Guide.docxQuestions for Panelists.docxHearing and Listening.mp4Disability, Culture & Language Accessibility.pptxShared Decision-Making Lecture.mp4Session Guide.docxStudent Guide.docxSession Evaluation Tool.doc [file mep_2374-8265.11396-s001.zip › H. Session Evaluation Tool.doc]

# STUDENT EVALUATION OF SESSION – Shared Decision Making at the Intersection of Disability, Culture, and Language Accessibility

**I.** Based on this week’s learning experiences (pre-session work, small-group activities, journal entries, clinical skills, etc.), **please indicate whether you feel that you are able to perform each of the learning objectives**

**Rating your confidence**

1. Definitely not
2. Probably not
3. Might or Might not
4. Probably yes
5. Definitely yes

| Learning Objectives | Confidence  **Before**  Session | Confidence  **After**  Session |
| --- | --- | --- |
| Identify that non-English language preference can act as a social determinant of health (for example in individuals who communicate using American Sign Language). | 1 2 3 4 5 | 1 2 3 4 5 |
| Apply elements of the medical and social models, practicing cultural humility, as appropriate in the context of Deaf culture. | 1 2 3 4 5 | 1 2 3 4 5 |
| Apply the key components of valid consent using a shared decision-making framework. | 1 2 3 4 5 | 1 2 3 4 5 |
| Describe the ability of all persons, regardless of disability to provide valid consent that reflects respect for self- determination. | 1 2 3 4 5 | 1 2 3 4 5 |

II. Please rate the educational **quality** of each of the following **center-based activities**:

*Poor*	*Excellent*

a) Pre-work	1	2	3	4	NA
b) Patient Scenario Discussion and Role-Play Exercise	1	2	3	4	NA
c) Family Panel	1	2	3	4	NA
III. Please rate the educational **quality** of each of the following **prework**:
d) Hearing and Listening video	1	2	3	4	NA
e) Shared Decision Making lecture video	1	2	3	4	NA
f) PowerPoint Terms and Clinical Tips	1	2	3	4	NA
IV. What did you like about the session?
V. What would you improve about the session?
